# Morphological and Molecular Characteristics of Choroid Plexus Epithelium in Aged Brains

**DOI:** 10.3390/ijms27052505

**Published:** 2026-03-09

**Authors:** Ryuta Murakami, Masaki Ueno

**Affiliations:** Department of Pathology and Host Defense, Faculty of Medicine, Kagawa University, 1750-1 Ikenobe, Miki-cho, Kita-gun, Takamatsu 761-0793, Kagawa, Japan

**Keywords:** choroid plexus, epithelial cells, E-cadherin, SPINT1, mitochondria

## Abstract

The choroid plexus (CP) has traditionally been regarded as a cerebrospinal fluid-producing structure; however, increasing evidence indicates that it functions as a dynamic regulatory interface involved in immune surveillance, metabolic homeostasis, and brain clearance. Neuroimaging studies consistently report CP enlargement across aging and diverse neurological and neuropsychiatric disorders, yet the underlying cellular mechanisms remain poorly integrated. In this review, we synthesize morphological, molecular, and imaging evidence to propose a sequential degenerative model of the CP epithelium. This model comprises: (1) regulated epithelial cell loss via apical extrusion, (2) compensatory hypertrophy of residual cells, (3) mitochondrial remodeling with oncocytic-like change, and (4) progressive blood–cerebrospinal fluid barrier dysfunction. At the molecular level, alterations in epithelial adhesion systems—particularly SPINT1-mediated protease regulation and E-cadherin–based adherens junction stability—may initiate epithelial instability. Hypertrophic epithelial cells exhibit increased mitochondrial burden, reflected by Tom20 expression, which may initially support metabolic adaptation but ultimately contribute to oxidative stress and functional decline. At the macroscopic level, the cumulative effects of cell loss, hypertrophy, and mitochondrial remodeling likely underlie CP enlargement detectable by magnetic resonance imaging. This framework positions CP enlargement as an imaging-visible manifestation of epithelial stress and provides a structural–molecular basis for interpreting CP alterations in brain aging and neurodegenerative disorders.

## 1. Introduction

The choroid plexus (CP) is a specialized epithelial structure located within the brain ventricles that plays a central role in cerebrospinal fluid (CSF) production and the maintenance of the brain microenvironment. Beyond its classical function as a CSF-secreting organ, the CP forms the blood–cerebrospinal fluid barrier (BCSFB), a highly regulated interface that controls molecular exchange between the peripheral circulation and the central nervous system [1,2,3,4,5].

In recent years, the CP has emerged as an active regulatory tissue involved in immune surveillance, metabolic homeostasis, and brain clearance mechanisms. Alterations in CP volume and function, especially CP volume enlargement, have been increasingly implicated in aging and a wide range of neurological disorders, including neurodegenerative and neuropsychiatric diseases [6,7,8,9]. However, despite accumulating molecular and imaging data, the cellular basis underlying CP dysfunction remains incompletely understood [10,11,12].

How age-related epithelial alterations translate into barrier breakdown, metabolic stress, and clinically detectable imaging changes has not been fully integrated into a unified framework. Recently, we reported key characteristics of CP epithelial cells, including the association between age and cell area and the presence of enlarged epithelial cells accompanied by increased mitochondrial content [13,14,15]. Although progress is being made in characterizing CP epithelial cells [16,17,18], the cause of CP enlargement remains unclear. This review aims to bridge this gap by synthesizing morphological, molecular, and imaging evidence, with a focus on epithelial cell–level changes in the CP [2,6,8,12].

In addition to synthesizing the broader literature, this review is informed by our recent work on (i) quantitative morphometry of CP epithelial cell loss and compensatory hypertrophy [13], (ii) serine protease inhibitor, Kunitz type 1 (SPINT1)-related regulation of epithelial adhesion and protease activity [14], and (iii) mitochondrial remodeling [15]. By integrating these lines of evidence, we propose a hypothesis of degenerative mechanism of the CP epithelial pathology across aging and neurological disease contexts.

## 2. Structure and Functional Characteristics of the CP Epithelium

### 2.1. Structure of the CP Epithelium-Formation of BCSFB-

The CP epithelium consists of a single layer of cuboidal to columnar epithelial cells interconnected by tight junctions and adherens junctions, collectively forming the BCSFB. Unlike the blood–brain barrier, which relies on endothelial tight junctions, the BCSFB is maintained primarily by epithelial junctional complexes. This structural organization allows selective transport of ions, nutrients, and signaling molecules from the bloodstream into the CSF while restricting uncontrolled diffusion [1,2,4].

**Figure 1:**
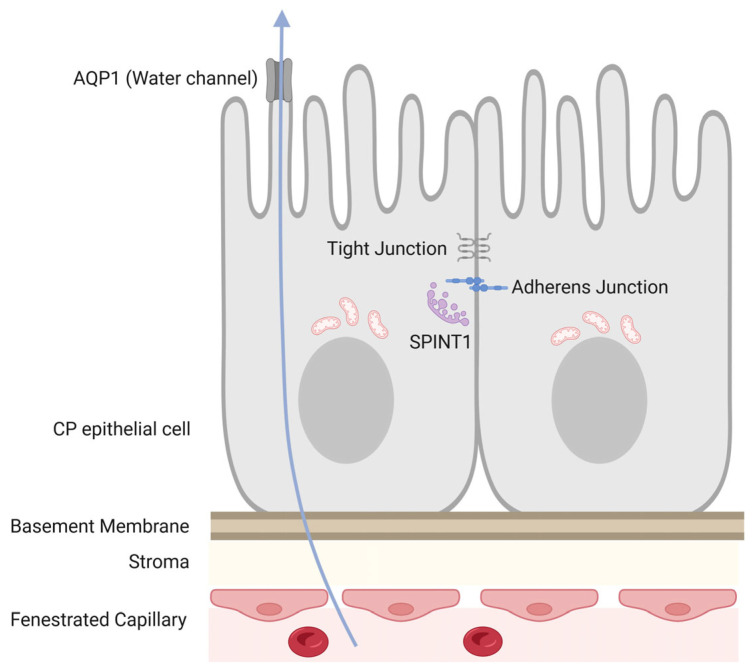
Structure and key molecular components of the CP epithelium. Schematic illustration of the CP epithelium and the BCSFB. CP epithelial cells form a single-layered epithelium sealed by tight junctions and cadherins–mediated adherens junctions. E-cadherin stability is regulated by epithelial protease–protease inhibitor systems, including SPINT1 (also known as HAI-1). The apical membrane is enriched in AQP1, supporting CSF secretion. CP epithelial cells contain abundant mitochondria; mitochondrial mass and distribution are commonly assessed by translocase of outer membrane 20 (Tom20). Beneath the epithelium, the basement membrane and stromal compartment overlie fenestrated capillaries, enabling selective blood-to-CSF transport under epithelial barrier control. Created in BioRender. Murakami, R. (2026) https://BioRender.com/16c99bn (accessed on 4 March 2026).

Previous studies have reported the presence of multiple cadherin subtypes, including N- and P-cadherin, at adherens junctions and lateral membranes of the CP epithelium [12,16,17]. In the present review, we place particular emphasis on E-cadherin to frame the discussion of junctional stability and barrier-relevant architecture. Tight junction components further reinforce barrier integrity, ensuring compartmentalization of the ventricular environment (Figure 1) [2,3,5].

### 2.2. Functional Characteristics of the CP Epithelium

CP epithelial cells actively regulate CSF composition through a coordinated network of transporters, channels, and pumps. Water transport is mediated predominantly by aquaporin-1 (AQP1), which is highly expressed on the apical membrane and is essential for CSF secretion [3,4,12]. In addition, various solute transporters support ionic balance, nutrient delivery, and waste removal [10,11,12].

These functions impose substantial energetic demands on CP epithelial cells. Accordingly, the cytoplasm of these cells is densely packed with mitochondria, reflecting a high basal metabolic rate. Maintenance of epithelial polarity, junctional integrity, and transport activity is therefore tightly coupled to mitochondrial function [12,17,18].

## 3. Morphological Changes in CP Epithelial Cells

**Figure 2:**
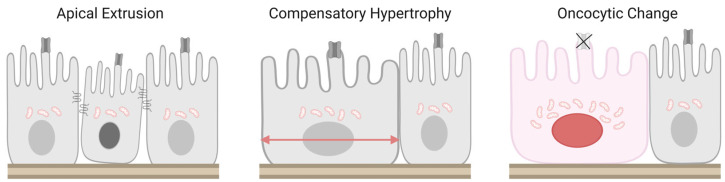
Sequential morphological changes in choroid plexus epithelial cells. Representative schematic of age- and disease-associated morphological changes in CP epithelial cells. Left: Apical extrusion, as shown in other epithelial cells [19,20], is supposed to be actively eliminated toward the ventricular lumen in damaged or senescent CP epithelial cells. The apical extrusion enables the preservation of epithelial continuity and barrier integrity. Middle: Compensatory hypertrophy, as shown in CP epithelial cells [13], characterized by lateral expansion of remaining epithelial cells to maintain coverage of the basement membrane following cell loss. Right: Oncocytic-like change, as shown in CP epithelial cells [13,15], induces marked cellular enlargement accompanied by increased mitochondrial accumulation, and results in an eosinophilic cytoplasm and nuclear rounding or displacement. This phenotype may reflect metabolic adaptation and increased mitochondrial dependence and may precede functional decline of the epithelium. Created in BioRender. Murakami, R. (2026) https://BioRender.com/yjqsn1h (accessed on 4 March 2026).

CP epithelial cells are expected to exhibit a spectrum of morphological alterations in association with aging and neurodegenerative disease. Representative changes include epithelial cell loss, compensatory hypertrophy of remaining cells, and oncocytic-like change [13,14,15]. Importantly, these features should not be regarded as independent phenomena but rather as interconnected stages within a continuous degenerative process that reflects progressive disruption of epithelial homeostasis (Figure 2) [19,20].

### 3.1. Epithelial Cell Loss and Extrusion Mechanisms

Loss of CP epithelial cells has long been recognized in aging brains and inflammatory conditions and has traditionally been described as epithelial “sloughing.” At the light microscopic level, detached epithelial cells are often observed floating in the ventricular lumen, frequently accompanied by eosinophilic cytoplasmic changes and nuclear condensation indicative of cellular degeneration [2,4].

In acute injury models, such as lipopolysaccharide exposure or ischemic insult, epithelial detachment can result in transient gaps within the epithelial layer and overt disruption of BCSFB integrity. However, in slowly progressive conditions such as aging and chronic neurodegeneration, the persistent presence of epithelial gaps would be incompatible with long-term barrier maintenance [2,5].

Recent advances in epithelial biology have established apical extrusion as a key mechanism for regulated epithelial cell removal [19,20]. In this process, damaged or senescent epithelial cells are actively expelled toward the luminal side while neighboring cells cooperatively reorganize through actomyosin ring formation. This coordinated response prevents exposure of the basement membrane and preserves epithelial continuity [19,20].

Accordingly, epithelial alterations in the aging CP may be interpreted as being consistent with controlled cell extrusion rather than purely passive sloughing, allowing gradual reduction in epithelial cell number while maintaining BCSFB integrity. Because direct in vivo evidence for extrusion in the CP remains limited, this interpretation is based primarily on histological observations together with established extrusion mechanisms in other epithelia. Nevertheless, under conditions of pronounced inflammaging or disruption of adhesion-regulatory pathways, this coordinated extrusion process may fail, resulting in overt barrier breakdown.

### 3.2. Compensatory Hypertrophy of Residual Epithelial Cells

As epithelial cells are eliminated through extrusion, the remaining cells are expected to expand their individual coverage of the basement membrane through compensatory hypertrophy. This response should not be viewed merely as passive gap filling but rather as an adaptive enlargement of each cell’s functional territory [13,21,22].

Quantitative morphometric analyses across multiple neurodegenerative disorders and aged controls demonstrate that epithelial cell area and axial dimensions do not differ significantly among diagnostic groups. Instead, these parameters show a robust positive correlation with age. These findings indicate that aging itself, rather than disease category, is the primary determinant of epithelial hypertrophy in the CP [13].

This biphasic pattern—progressive reduction in cell number accompanied by enlargement of surviving cells—suggests that epithelial maintenance relies on compensatory mechanisms that redistribute functional burden across fewer cells. While initially effective, such compensation inevitably increases metabolic demand at the single-cell level [13,23,24].

### 3.3. Oncocytic-like Change and Morphological Context

With advancing age, subsets of CP epithelial cells acquire an oncocytic-like morphology, characterized by enlarged eosinophilic cytoplasm, increased mitochondrial content, and nuclear rounding or displacement. Oncocytic-like change is not unique to the CP but represents a common age-associated phenomenon observed across multiple epithelial tissues subjected to chronic metabolic stress [23,24]. In the human CP, aging has been reported to be associated with epithelial atrophy and decreased cell height, total volume, and apical microvillus length [25]. In addition, aged CP epithelial cytoplasm becomes enriched in Biondi ring tangles and lipofuscin deposits, findings that are also observed in the CP of patients with Alzheimer’s disease [25,26].

Within this framework, oncocytic-like change in the CP is best interpreted as a morphological manifestation of metabolic overload superimposed on compensatory hypertrophy. Rather than representing a discrete pathological entity, it marks a transitional state in which adaptive capacity is progressively exhausted [21,22]. Commentary studies addressing morphometric analyses have further emphasized the importance of measuring context, including tissue preparation and analytical scale, in interpreting epithelial morphology [21,22]. These discussions underscore that apparent discrepancies across studies are not contradictory but instead highlight the multilayered nature of epithelial degeneration, spanning ultrastructural, cellular, and tissue-level dimensions.

### 3.4. Age-Related and Disease-Associated Vulnerability of the CP

With aging, the CP is expected to undergo structural and functional remodeling characterized by epithelial irregularity, altered junctional organization, and changes in mitochondrial morphology. In addition, chronic low-grade inflammation (inflammaging) further compromises epithelial stability, predisposing the BCSFB to dysfunction [27,28,29].

At the systems level, these cellular alterations are increasingly reflected in neuroimaging studies such as CP enlargement and microstructural changes detectable by magnetic resonance imaging (MRI) [6,7,8,9]. Such findings have been reported across multiple conditions, including normal aging [6,28,29,30], inflammation [31], neurodegenerative diseases [7,32,33,34], and psychiatric disorders [8,35,36,37], suggesting that CP alterations may represent a shared substrate of brain aging rather than a disease-specific phenomenon.

Together, these observations underscore the importance of understanding CP epithelial pathology at the cellular level. A detailed examination of epithelial morphology, adhesion, and metabolism is therefore essential for elucidating how age-related changes in the CP contribute to barrier dysfunction and brain pathology.

## 4. Adhesion Molecules and Protease Regulation: SPINT1 and the Adhesive Architecture of the CP Epithelium

### 4.1. E-Cadherin-Centered Adhesion Architecture

Adherens junctions in CP epithelial cells are primarily organized by E-cadherin, providing the mechanical linkage required for stable cell–cell adhesion and continuity of the epithelial layer. Membranous E-cadherin localization is therefore central to maintaining barrier-relevant architecture across the epithelium. A reduction in E-cadherin expression or altered subcellular localization may weaken intercellular adhesion, thereby increasing susceptibility to epithelial detachment and sloughing, particularly under aging- or disease-associated stress [2,5].

### 4.2. SPINT1 Expression and Function

SPINT1 (also known as hepatocyte growth factor activator inhibitor type 1; HAI-1) is a membrane-associated inhibitor containing two Kunitz domains that controls pericellular serine proteases such as matriptase [38]. By modulating local proteolysis at the epithelial surface, SPINT1 contributes to maintenance of epithelial integrity and protection from excessive protease-driven injury. The prominent expression of SPINT1 in the CP epithelium supports the concept that this tissue relies on tight protease regulation as a self-protective mechanism to preserve epithelial structure [14,25].

### 4.3. The SPINT1-E-Cadherin-SIP1 Axis and Sloughing

SPINT1 can preserve epithelial adhesion by restraining matriptase activity, thereby limiting E-cadherin cleavage and junctional destabilization [38]. When this regulatory layer is weakened, adhesion failure may synergize with transcriptional repressors such as Smad-interacting protein 1 (SIP1), which can downregulate E-cadherin and promote epithelial instability. SPINT1 also regulates epithelial-to-mesenchymal transition (EMT), suggesting that its dysregulation may contribute to epithelial loss and mesenchymal proliferation including fibrosis [39]. In this context, regulated extrusion would be expected to remove compromised cells while maintaining tissue continuity; disruption of this balance could shift toward pathological sloughing accompanied by barrier dysfunction or, conversely, delayed removal of dying cells with secondary inflammatory consequences [14].

## 5. Mitochondrial Dysfunction and Tom20: Metabolic Background of Hypertrophy and Oncocytic-like Change

### 5.1. Tom20 and the Mitochondrial Vulnerability of CP Epithelial Cells

Tom20 is a core component of the translocase of the outer mitochondrial membrane complex and is widely used as an immunohistochemical marker reflecting mitochondrial mass and distribution. Tom20 plays a role as an initial receptor for importing cytosolically synthesized proteins into mitochondria [31,40]. CP epithelial cells support energy-demanding processes, including CSF production and active solute transport, and therefore contain abundant mitochondria. Consequently, mitochondrial dysfunction in these cells is likely to have a direct and disproportionate impact on CP function, rendering the epithelium metabolically vulnerable during aging and disease [15,31,40].

### 5.2. Quantitative Tom20 Immunohistochemistry: Association Between Cellular Hypertrophy and Mitochondrial Density

To characterize mitochondrial alterations in the CP epithelium, Tom20 immunohistochemistry was subjected to quantitative image analysis. Across neurodegenerative disease cases and age-matched controls, mitochondrial density did not differ significantly among diagnostic groups. In contrast, a significant positive correlation was observed between epithelial cell area and Tom20-positive signal intensity, indicating that enlarged epithelial cells harbor a greater mitochondrial burden [13,15]. These findings suggest that mitochondrial expansion is not a disease-specific phenomenon but rather a common adaptive response accompanying epithelial hypertrophy. In this context, hypertrophy appears to be coupled with mitochondrial remodeling aimed at sustaining increased energetic demands imposed on enlarged cells [15,29,40].

### 5.3. Oncocytic-like Change, AQP1 Alterations, and the Limits of Metabolic Adaptation

Detailed inspection of Tom20-stained sections revealed focal populations of markedly enlarged epithelial cells exhibiting oncocytic-like morphology and intense Tom20 immunoreactivity. Dual immunofluorescence analyses further demonstrated that, in a subset of hypertrophic cells, strong Tom20 positivity was accompanied by reduced or absent expression of AQP1, a key mediator of water transport and CSF secretion [3,12,15].

This dissociation suggests that increased mitochondrial mass does not necessarily translate into preserved epithelial function. Instead, oncocytic-like change may represent a state in which metabolic adaptation persists despite declining transport capacity, reflecting a functional imbalance between energy production and specialized epithelial activity [15,40].

### 5.4. Mitochondrial Stress and Progressive BCSFB Dysfunction

Alterations in Tom20 expression reflect not only quantitative mitochondrial expansion but also qualitative deterioration of mitochondrial function. Mitochondrial impairment leads to reduced ATP availability and excessive generation of reactive oxygen species (ROS), both of which compromise the maintenance of tight junctions and adherens junctions [3,12]. Excessive ROS released from dysfunctional mitochondria can directly modify or destabilize junctional proteins, promoting increased permeability of the BCSFB. Thus, mitochondrial expansion that initially serves as a compensatory mechanism may, beyond a critical threshold, become a primary driver of oxidative stress and epithelial barrier failure [12,29,31]. This self-amplifying cycle of mitochondrial stress and barrier disruption provides a mechanistic link between cellular hypertrophy, oncocytic-like change, and irreversible CP dysfunction [31].

## 6. Imaging Correlates of CP Alterations: Insights from Magnetic Resonance Imaging

Advances in neuroimaging have enabled noninvasive assessment of the CP in vivo, providing important insights into its structural and functional alterations during aging and disease. Among these modalities, magnetic resonance imaging (MRI) has emerged as a powerful tool for quantifying CP volume [6,7,8,9].

### 6.1. Choroid Plexus Enlargement Across Aging and Neurological Disorders

Multiple MRI studies have demonstrated that CP volume increases with aging in healthy individuals [6,9,30]. This enlargement is not restricted to pathological states but appears to represent a gradual and continuous process throughout the adult lifespan. Importantly, similar CP enlargement has been reported across a wide range of neurological disorders, including Alzheimer’s disease, multiple sclerosis, and schizophrenia-spectrum disorders [7,8,9,32,33,34,35,36,37].

In multiple sclerosis, CP enlargement has been associated with lesion expansion, white matter atrophy, and disease activity, suggesting a link between CP alterations and chronic neuroinflammation [7,33]. In neuropsychiatric conditions, increased CP volume has been correlated with cognitive impairment, inflammatory markers, and structural brain changes, further supporting the notion that CP enlargement reflects systemic and central nervous system-wide pathological processes rather than disease-specific abnormalities [35,36,37].

### 6.2. Linking MRI Findings to Epithelial Pathology

While MRI-based CP enlargement is a robust and reproducible finding, its cellular and molecular underpinnings have remained unclear. Integrating imaging data with histopathological observations suggests that CP enlargement may arise from a combination of epithelial hypertrophy, oncocytic-like change, and stromal remodeling, rather than simple hyperplasia [13,15,27].

As discussed in preceding sections, aging is associated with progressive epithelial cell loss accompanied by compensatory hypertrophy of remaining cells. Such cellular enlargement, together with increased mitochondrial content and metabolic stress, may collectively contribute to macroscopic increases in CP volume detectable by MRI. Notably, these changes can occur even in the absence of overt increases in epithelial cell number, providing a plausible explanation for imaging–histology discrepancies [13,21,22].

### 6.3. Implications for CSF Dynamics and Brain Clearance

Beyond structural considerations, CP enlargement may have important functional implications. Alterations in epithelial transport capacity, junctional integrity, and mitochondrial function are likely to affect CSF production and composition. Disruption of these processes may, in turn, influence CSF flow dynamics and brain clearance pathways [2,5]. Recently, an increasing number of studies have highlighted the importance of the glymphatic system—a major pathway for clearance of waste products from the brain—in relation to CSF flow dynamics [41,42,43]. In addition, one study reported that glymphatic function and CP volume are associated with systemic inflammation and oxidative stress in major depressive disorder [44]. Emerging evidence suggests that impaired CSF dynamics contribute to reduced clearance of metabolic waste and inflammatory mediators from the brain parenchyma. In this context, MRI-detectable CP alterations may serve as indirect markers of compromised BCSFB function and impaired brain homeostasis [6,7,8,9]. These findings suggest that CP imaging may provide a translational biomarker.

## 7. A Hypothesized Degenerative Mechanism of the CP Epithelium: Integration of Morphological, and Imaging Findings

**Figure 3:**
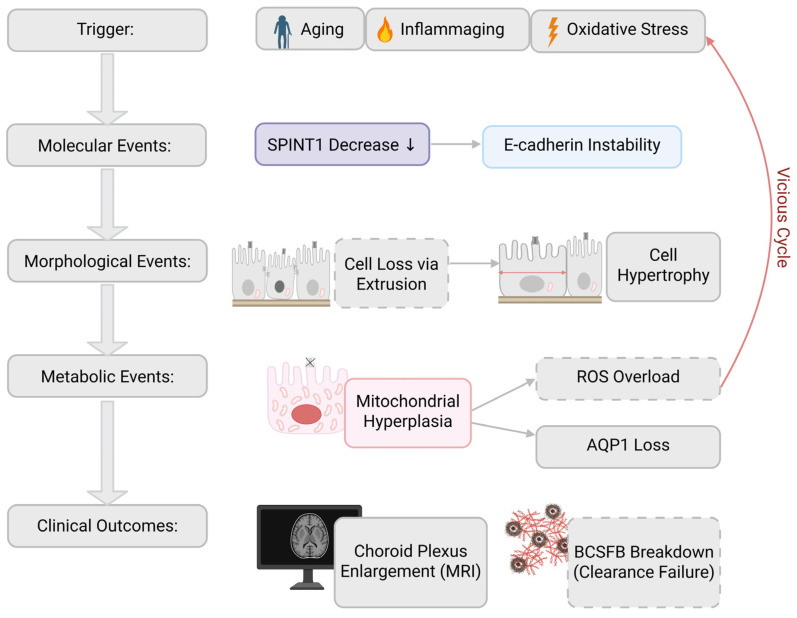
A hypothesized degenerative mechanism of the CP epithelium. According to recent findings—including inflammation-associted oxidative stress [31], age-associated epithelial hypertrophy [13], decreased SPINT1 and E-cadherin instability [14], mitochondrial expansion (oncocytic-like change) with AQP1 loss [15], and CP enlargement on MRI in aging and neurodegenerative diseases [6,7,8,9,32,33,34,35,36,37]—a degenerative mechanism of CP epithelial cells can be proposed, incorporating plausible intermediate events such as cell loss, ROS overload, and BCSFB breakdown (i.e., impaired clearance of interstitial fluid). The proposed mechanism integrating molecular, morphological, and imaging findings is shown. Aging, inflammaging, and oxidative stress act as upstream triggers that may lead to molecular alterations such as reduced SPINT1 expression and destabilization of E-cadherin–mediated adherens junctions. These changes may promote epithelial cell loss through extrusion. To compensate for reduced cell number, remaining epithelial cells undergo hypertrophy, increasing their territorial coverage. Hypertrophic cells exhibit mitochondrial expansion (oncocytic-like change), often accompanied by increased Tom20 expression, ROS overload, and downregulation or loss of AQP1 expression. Excessive mitochondrial stress further exacerbates oxidative damage, forming a vicious cycle that accelerates epithelial dysfunction. At the clinical level, these cumulative alterations manifest as CP enlargement detectable by MRI and are associated with BCSFB dysfunction and impaired brain clearance mechanisms. Specific data were circled by solid lines, whereas inferred descriptions were circled by dotted lines. Oxidative stress is associated with inflammation, whereas cell hypertrophy is associated with aging. In addition, BCSFB breakdown is associated with CSF dynamics dysorder, whereas CP enlargement detected by MRI is associated with aging and neurodegeneration. Created in BioRender. Murakami, R. (2026) https://BioRender.com/72cit67 (accessed on 4 March 2026).

Integrating the findings discussed above, accumulating evidence supports the notion that CP epithelial cells undergo a coordinated degenerative cascade under conditions of aging and chronic neurological disease (Figure 3) [2,6].

At the initial stage, inflammaging and chronic oxidative stress are proposed to contribute to molecular alterations in epithelial adhesion systems, potentially involving dysregulated SPINT1 expression (e.g., reduction in vulnerable cells or compensatory upregulation in hypertrophic cells [14]) and destabilization of E-cadherin mediated adherens junctions. Together with alterations in tight junction components, these changes may render the BCSFB structurally vulnerable [19,20,25].

Subsequently, epithelial cells that fail to maintain stable adhesion may be removed via a process consistent with apical extrusion, in which damaged or senescent cells are actively expelled toward the ventricular lumen while epithelial continuity is preserved. In the CP, direct in vivo evidence for extrusion remains limited; therefore, we interpret histological observations of ventricularly oriented epithelial shedding/detachment as compatible with extrusion rather than purely passive sloughing. As a compensatory response to cell loss, the remaining epithelial cells undergo hypertrophy, expanding their individual territorial coverage on the basement membrane. Morphometric analyses indicate that this hypertrophic response correlates more strongly with aging than with specific neurodegenerative diagnoses, suggesting that it represents a common adaptive process rather than a disease-specific phenomenon [13]. Hypertrophic epithelial cells exhibit a marked increase in mitochondrial content, reflected by elevated Tom20 expression, consistent with mitochondrial remodeling and metabolic adaptation. It remains to be clarified whether oncocytic-like change directly impairs multiple epithelial functions, including BCSFB integrity and AQP1-mediated water transport. Nevertheless, it is plausible that progressive epithelial dysfunction accelerates CP degeneration [13,15,25]. At the macroscopic level, the cumulative effects of epithelial cell loss, hypertrophy, and oncocytic-like change manifest as CP enlargement and structural alterations detectable by MRI. These imaging findings likely reflect underlying barrier dysfunction and impaired CSF dynamics, linking cellular pathology to clinically observable phenotypes [2,19].

Collectively, this hypothesized degenerative mechanism reframes the CP not as a passive structure undergoing age-related change but as a dynamic regulatory tissue whose adaptive responses ultimately shape the trajectory of brain aging and neurodegenerative disease progression [13,14,15].

## 8. Comparative Perspective: CP Versus Other Hypertrophic Epithelia

### 8.1. Shared Principles of Compensatory Epithelial Hypertrophy Under Chronic Stress

Chronic inflammatory and metabolic stress frequently elicit characteristic adaptive responses in epithelial tissues, marked by progressive cell loss accompanied by compensatory hypertrophy of surviving cells. Rather than representing simple hyperplasia, this process reflects a structural and functional reorganization aimed at preserving tissue-level function despite declining cellular reserves, and has been described across diverse epithelial systems exposed to sustained stressors such as aging, chronic inflammation, or persistent metabolic challenge [45]. A defining feature of this adaptive response is disproportionate enlargement of individual epithelial cells, often with increased organelle content, particularly mitochondria, leading to escalated metabolic demands and vulnerability to oxidative stress and bioenergetic imbalance [46,47].

Epithelial homeostasis also involves regulated mechanisms of cell removal that preserve barrier integrity. Live cell extrusion at the apical surface selectively removes aged or damaged cells without disrupting the continuity of the epithelium, constituting a conserved strategy for maintaining barrier function under stress [48,49]. When chronic stress persists, compensatory hypertrophy may exhaust cellular capacity, with enlarged cells approaching a metabolic threshold beyond which functional efficiency declines and barrier properties are compromised [47]. Under such conditions, mitochondrial accumulation and altered organelle morphology may reflect a late-stage adaptive failure rather than sustained functional compensation [46].

This conceptual framework allows interpretation of oncocytic-like change as an extreme manifestation of chronic stress–induced hypertrophy. Oncocytic phenotypes, characterized by abundant mitochondria, have been documented in multiple epithelial tissues subject to persistent stress, including thyroid follicular epithelium and renal epithelia where they often coincide with functional decline and increased oxidative burden [50,51]. Such transformations likely represent a convergent, stress-driven cellular phenotype rather than a tissue-specific anomaly. The CP epithelium conforms to this general paradigm. As a specialized epithelium responsible for CSF secretion and maintenance of BCSFB function, it is subjected to continuous inflammatory cues and systemic metabolic changes associated with aging. It is likely that epithelial cell loss followed by hypertrophy of residual cells may initially support CSF dynamics. However, progressive enlargement and metabolic strain can predispose the epithelium to junctional instability and functional decline, setting the stage for subsequent barrier dysfunction and macroscopic structural alterations consistent with in vivo imaging observations.

### 8.2. Oncocytic-like Change as a Convergent Epithelial Phenotype

Oncocytic-like change has traditionally been described in organ-specific contexts, most notably in the thyroid follicular epithelium, renal parenchyma, and salivary gland ducts, where it is characterized by marked cellular enlargement and excessive mitochondrial accumulation. Although often discussed in relation to neoplastic processes, increasing evidence suggests that oncocytic features can emerge more broadly as a cellular response to chronic metabolic and oxidative stress, independent of overt tumorigenesis [46,47,50]. From this perspective, oncocytic-like change may be regarded as a convergent epithelial phenotype reflecting prolonged adaptive pressure rather than a tissue-restricted abnormality.

In classical oncocytic epithelia, such as Hürthle cell lesions of the thyroid, mitochondrial proliferation is frequently accompanied by impaired respiratory efficiency, increased reactive oxygen species production, and accumulation of mitochondrial DNA alterations [46,50]. Similar mitochondrial-rich phenotypes have been reported in renal oncocytosis and related renal epithelial conditions, where abundant mitochondria coexist with altered metabolic profiles and limited functional reserve [51]. Across these tissues, mitochondrial enrichment appears to represent a compensatory response to sustained energetic demand that ultimately becomes maladaptive.

Importantly, mitochondrial abundance should not be equated with preserved or enhanced function. Instead, oncocytic-like cells often exhibit a paradoxical state of “mitochondrial excess with functional inefficiency,” in which increased organelle mass fails to offset declining bioenergetic capacity [46,47]. This concept is particularly relevant when interpreting markers of mitochondrial content, such as Tom20, which reflect organelle abundance but not necessarily respiratory competence. Within this framework, increased Tom20 expression may signify accumulated metabolic stress rather than successful adaptation [15].

The CP epithelium can be interpreted within this broader epithelial paradigm. Recent morphometric and molecular analyses indicate the presence of enlarged epithelial cells with increased mitochondrial markers in the aging CP, consistent with a stress-driven hypertrophic trajectory rather than a distinct pathological entity. Thus, oncocytic-like remodeling of the choroid plexus epithelium is best understood as part of a shared epithelial response to chronic metabolic and inflammatory stress, adapted to the unique secretory and barrier functions of this tissue.

What distinguishes the CP is not the occurrence of oncocytic-like remodeling itself, but the extent to which cumulative epithelial hypertrophy translates into macroscopic structural changes. In contrast to other epithelia, where such remodeling remains largely confined to microscopic observations, CP enlargement becomes detectable at the organ level by neuroimaging, providing a unique in vivo window into epithelial stress responses.

### 8.3. Why the CP Is Uniquely Imaging-Visible

Among epithelial tissues exhibiting compensatory hypertrophy and oncocytic-like remodeling, the CP is distinctive in that cumulative cellular alterations manifest as macroscopic structural changes detectable by neuroimaging. While similar stress-driven epithelial remodeling in other organs is largely appreciated through histological or ultrastructural analyses, changes in the CP frequently translate into measurable volumetric enlargement on magnetic resonance imaging. This unique imaging visibility likely reflects the anatomical organization, functional demands, and spatial constraints of the CP.

The CP epithelium performs dual and energetically demanding roles, combining continuous cerebrospinal fluid secretion with maintenance of a selective BCSFB. Unlike many peripheral epithelia, it lacks a large regenerative reserve and is composed of a highly folded epithelial sheet surrounding a vascular core within a confined ventricular space. Under conditions of aging-associated inflammation and metabolic stress, regulated epithelial cell loss followed by compensatory hypertrophy of surviving cells may initially preserve cerebrospinal fluid dynamics. However, progressive enlargement of individual epithelial cells, together with increased mitochondrial burden, inevitably alters tissue geometry and volume.

Importantly, volumetric enlargement of the CP does not necessarily imply enhanced functional capacity. Rather, imaging-detectable hypertrophy may represent the cumulative outcome of epithelial compensation approaching its metabolic limits. As hypertrophy progresses, escalating bioenergetic demand, oxidative stress, and junctional instability are likely to compromise barrier integrity and secretory efficiency. Thus, macroscopic enlargement may reflect a late-stage adaptive response rather than sustained functional augmentation.

Neuroimaging studies consistently reporting CP enlargement across aging and diverse neurological and neuropsychiatric disorders can therefore be interpreted within this cellular framework. Rather than representing disease-specific pathology, choroid plexus enlargement may serve as an integrated marker of epithelial stress, capturing the net effect of cell loss, compensatory hypertrophy, and mitochondrial remodeling over time. In this sense, the CP provides a rare opportunity to link epithelial cell–level degeneration with in vivo imaging readouts.

Taken together, the imaging visibility of the CP is not an incidental observation but a direct consequence of its unique epithelial architecture and functional burden. This property positions choroid plexus volume as a potential surrogate marker of epithelial health and aging-related stress, bridging microscopic cellular alterations with macroscopic neuroimaging findings.

## 9. Conclusions and Future Perspectives

In this review, we integrated morphological, molecular, and imaging evidence to propose a hypothesized degenerative mechanism of the CP epithelium in aging and neurological disease. Rather than representing isolated or disease-specific abnormalities, the changes observed in CP epithelial cells appear to follow an ordered cascade involving cell loss, compensatory hypertrophy, mitochondrial remodeling, and eventual barrier dysfunction [2,6,13,15,19].

Traditionally, the CP has been regarded primarily as a CSF-producing structure. However, accumulating evidence indicates that it functions as a dynamic regulatory interface that contributes to immune surveillance, metabolic homeostasis, and brain clearance mechanisms. The present synthesis highlights that age-related epithelial degeneration is not a passive process but reflects repeated cycles of adaptive responses and their eventual failure [6,19,23].

A key insight emerging from morphometric analyses is that epithelial hypertrophy correlates more strongly with aging than with specific neurodegenerative diagnoses. This finding suggests that CP pathology may represent a shared substrate of brain aging, upon which disease-specific processes are superimposed. At the cellular level, compensatory hypertrophy is accompanied by mitochondrial expansion and oncocytic-like changes, reflecting increased energetic demand and metabolic stress [13,15,19,30].

At the systems level, these cumulative epithelial alterations are increasingly detectable by MRI, most prominently as CP enlargement and structural abnormalities. Such imaging findings should not be viewed as epiphenomena but rather as macroscopic reflections of microscopic epithelial stress and barrier dysfunction, integrating the net effects of cell loss, compensatory hypertrophy, and mitochondrial remodeling. These changes may have important implications for CSF dynamics, glymphatic clearance, and neuroinflammation [2,6,19,23].

Looking forward, several challenges remain. Longitudinal human studies are required to delineate the temporal progression of epithelial degeneration and to clarify its relationship to cognitive decline and disease onset. In parallel, experimental approaches targeting epithelial adhesion pathways, mitochondrial homeostasis, or oxidative stress may provide new opportunities to preserve BCSFB function. Finally, it is likely that the integration of molecular pathology with advanced neuroimaging holds promise for establishing the choroid plexus as a diagnostic and therapeutic entry point in brain aging and neurodegenerative disorders.

Collectively, the framework proposed here redefines the CP as an active participant in brain aging rather than a passive bystander. By positioning epithelial degeneration at the intersection of cellular adaptation, metabolic stress, and barrier failure, this model offers a conceptual basis for future mechanistic studies and translational strategies [2,6,19]. CP abnormalities may contribute to the deterioration of cognitive function, as the CP is located close to the hippocampus.

## Data Availability

No new data were created or analyzed in this study.

## Abbreviations

The following abbreviations are used in this manuscript:

AQP1	Aquaporin-1
ATP	Adenosine triphosphate
BCSFB	Blood–cerebrospinal fluid barrier
CP	Choroid plexus
CPE	Choroid plexus epithelium
CSF	Cerebrospinal fluid
HAI-1	Hepatocyte growth factor activator inhibitor type 1
MRI	Magnetic resonance imaging
ROS	Reactive oxygen species
SIP1	Smad-interacting protein 1
SPINT1	Serine protease inhibitor Kunitz type 1
TOM20	Translocase of outer mitochondrial membrane 20
